# Supplementary material for: Assessing the generalization capabilities of TCR binding predictors via peptide distance analysis
Source: PLoS One. 2025 May 20;20(5):e0324011. doi: 10.1371/journal.pone.0324011 (PMC12091837; doi:10.1371/journal.pone.0324011)
Supplement: S1 File — Supplementary Tables and Figures. Supplementary figures and tables referenced in the main text, including TCR-pMHC complex diagrams, additional model results, and extended correlation tables. See also Supplementary Sects 2.1–2.3 for details (PDF) [file pone.0324011.s001.pdf]

Supplementary Tables and Figures

TCR-pMHC Complex Overview

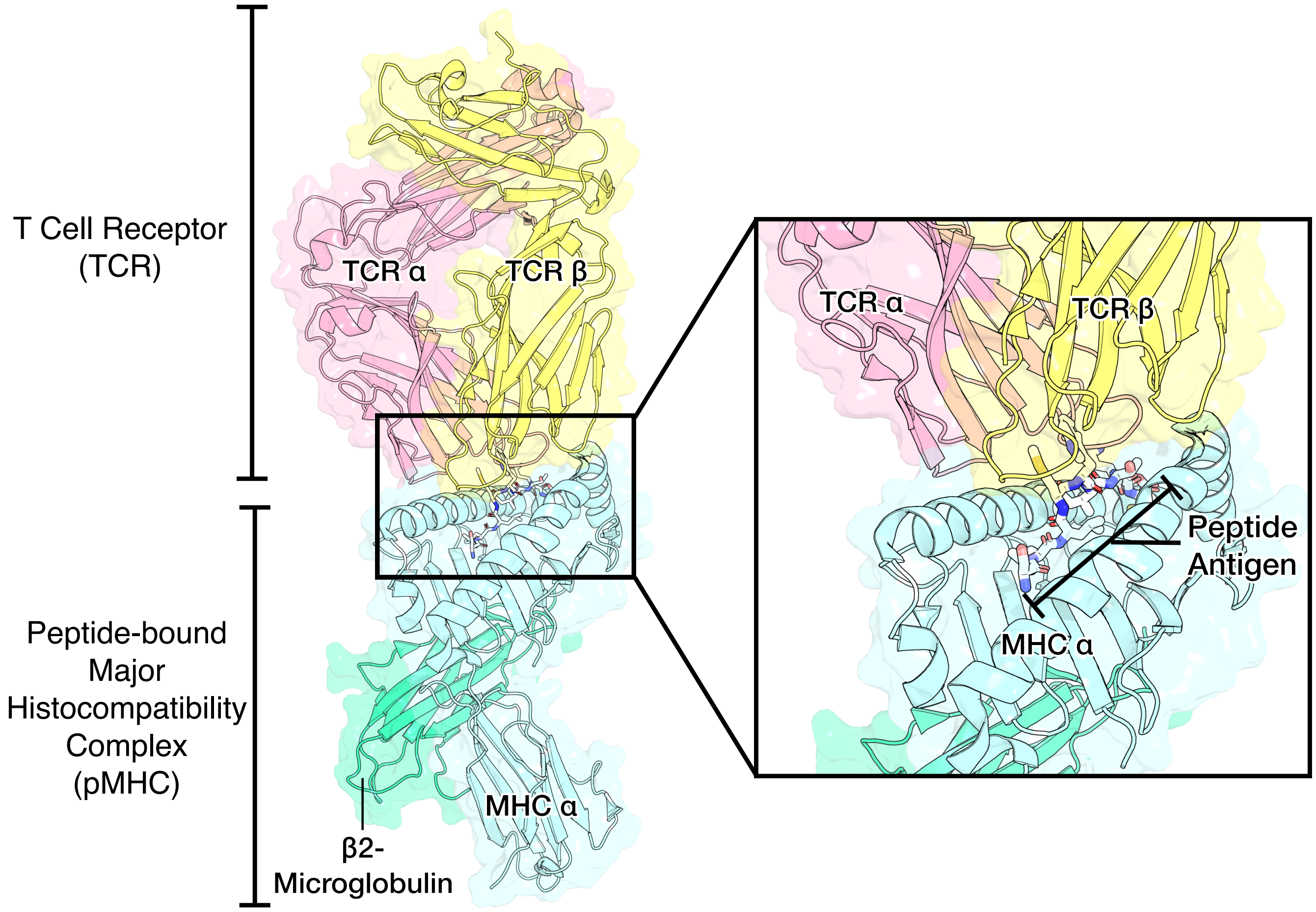

Figure S1. Overview of the TCR-pMHC Complex. The TCR is composed of an α and β chain which directly interact with the peptides. The MHC binds the peptide. Class 1 MHCs (pictured above) are composed of an α chain and a β2-Microglobulin. Class 2 MHCs, on the other hand, are composed of an α and β chain.

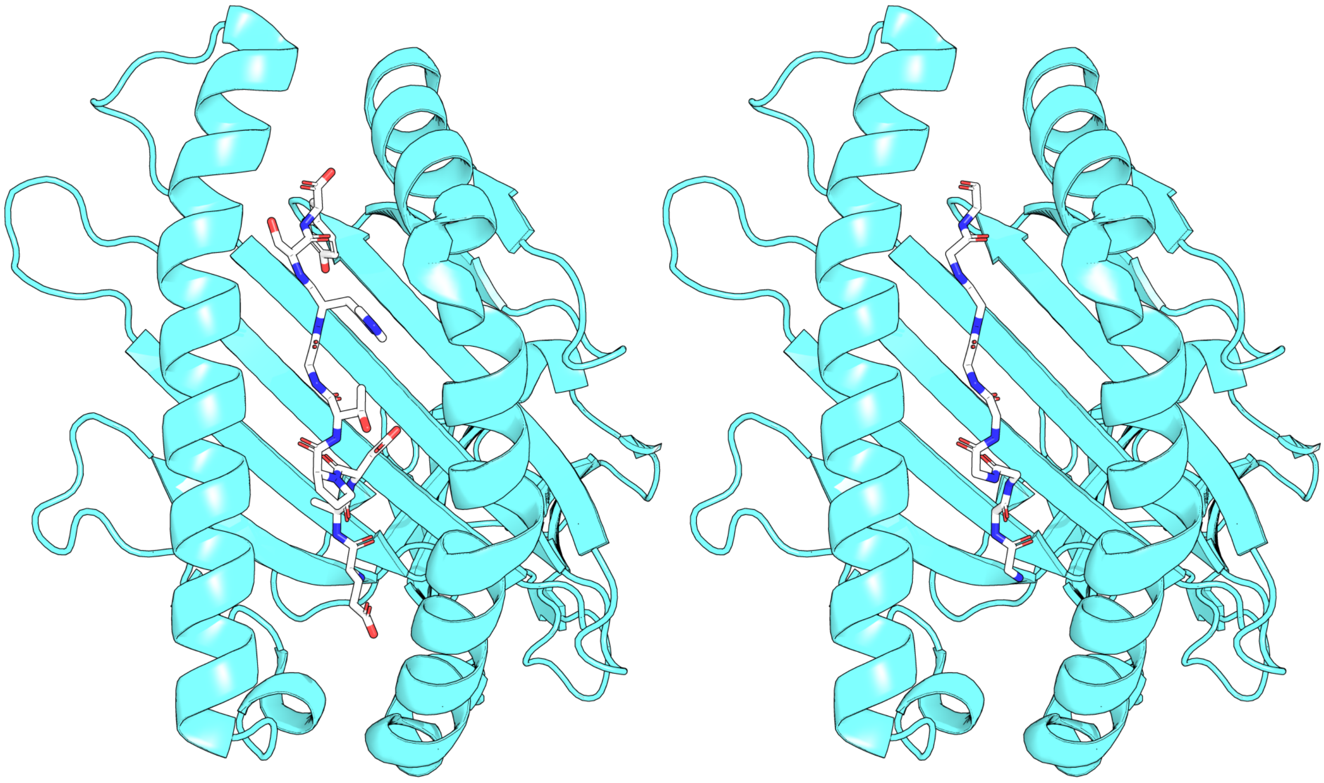

Figure S2. Top view of the HLA (cyan) with the peptide, with side chain atoms (left) and backbone only (right).

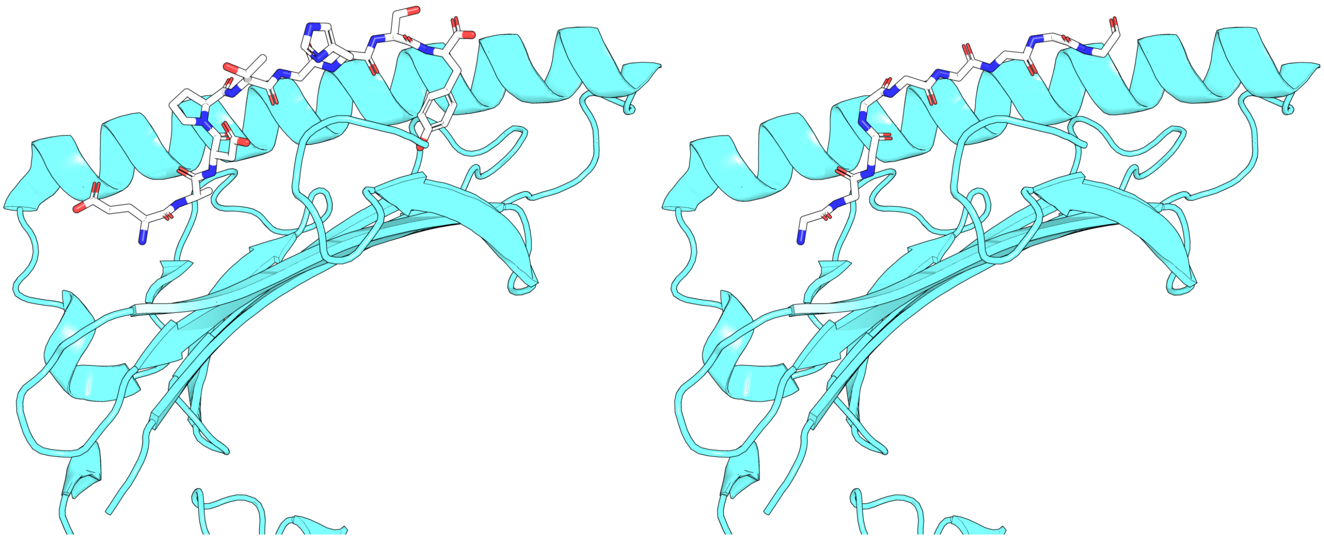

Figure S3. Side view of the HLA (cyan) with the peptide, with side chain atoms (left) and backbone only (right).

1 TCR-PMHC MODELS IMPLEMENTATION

We used the TCRModels library available on GitHub at this link to run Attentive Variational Information Bottleneck (AVIB) [Grazioli et al., 2022b], NetTCR-2.0 Jurtz et al. [2018], NetTCR-2.2 Jensen and Nielsen [2024], ERGO II (with pre-trained TCR autoencoder), and ERGO II-LSTM Springer et al. [2021b].

All models were trained 5 times, with 5 different random seeds. 300 epochs with early stopping patience of 50 epochs were employed. We used optimal parameters for each of these models provided in the corresponding publications.

1.1 Splits Peptide Numbers

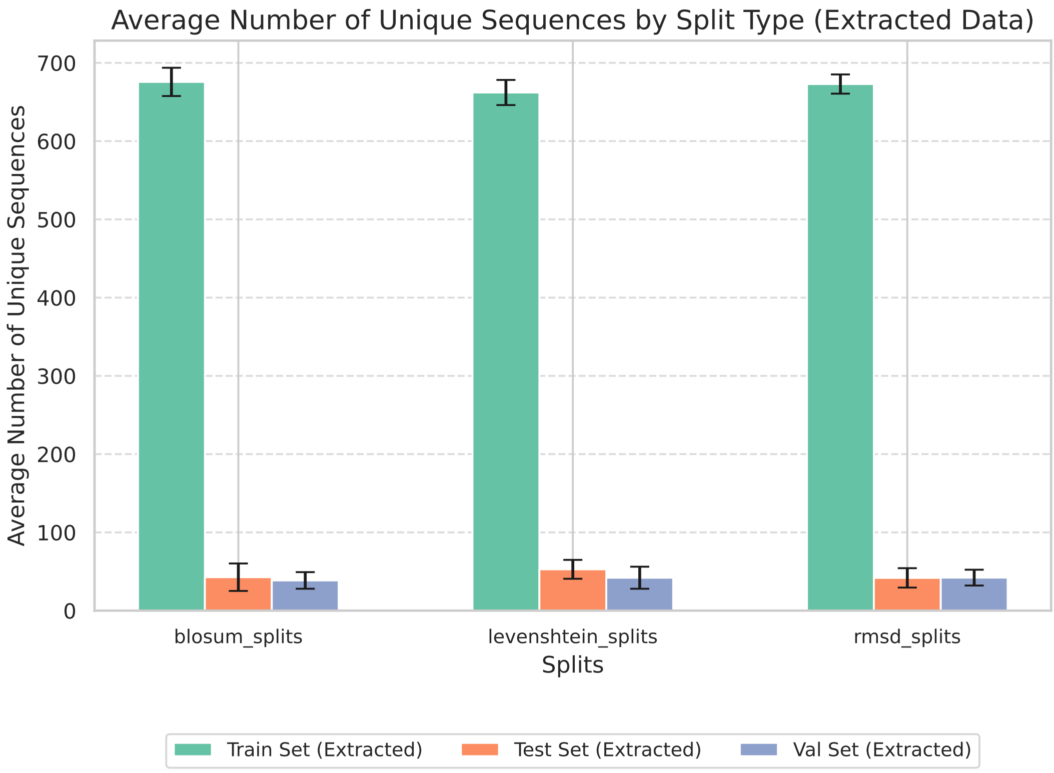

Figure S4. Number of peptides per split type in the Distance Split.

2 PERFORMANCE SCORES TCR-PMHC MODELS

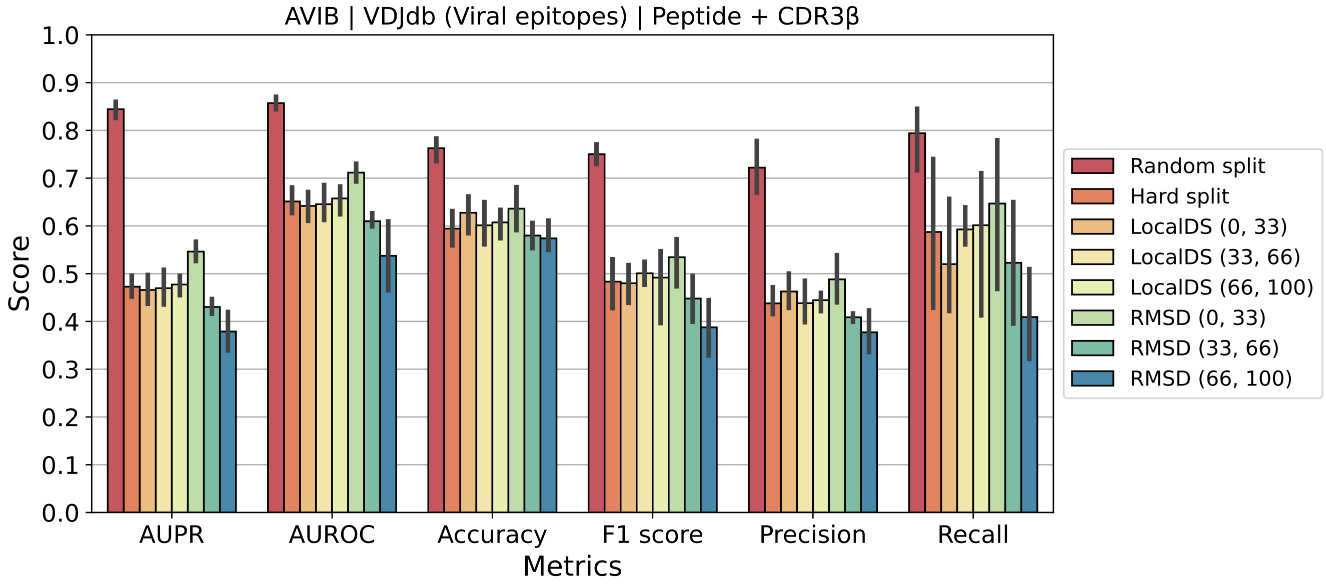

Figure S5. Performance metrics for the AVIB model [Grazioli et al., 2022a].

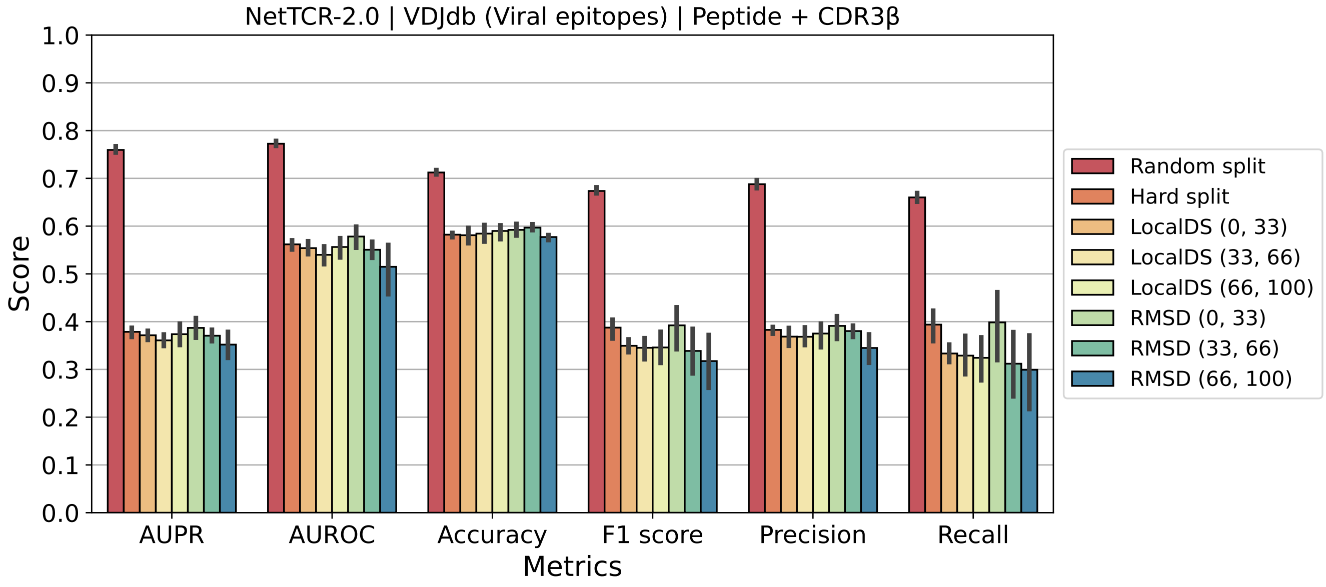

Figure S6. Performance metrics for the NetTCR-2.0 model [Jurtz et al., 2018].

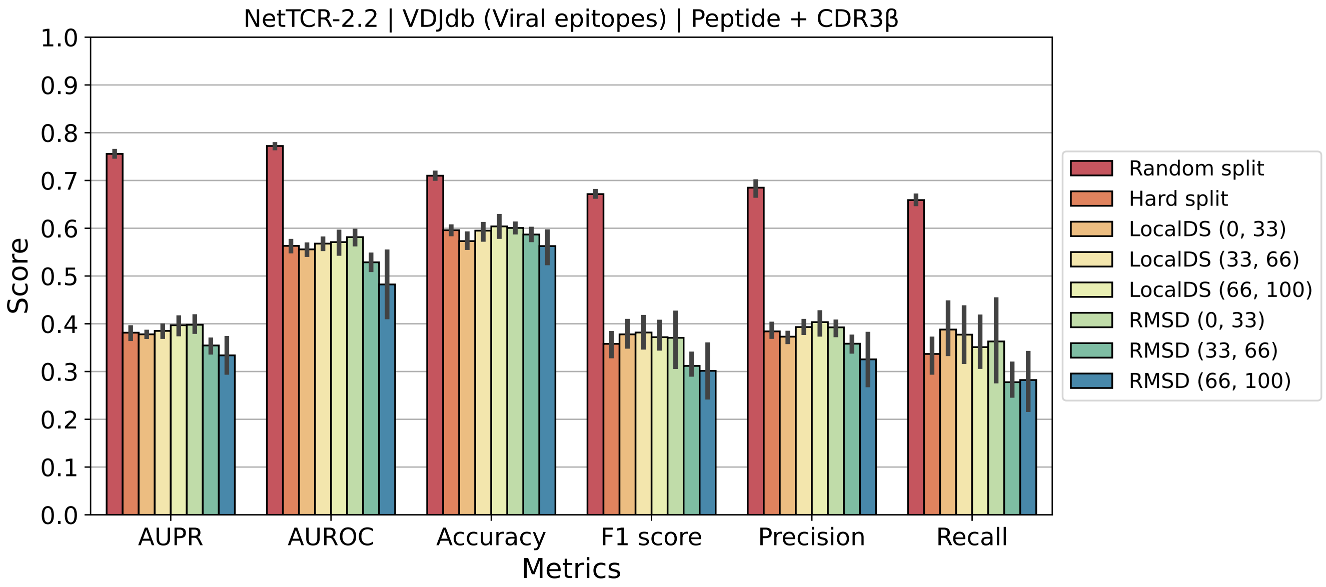

Figure S7. Performance metrics for the NetTCR-2.2 model [Jensen and Nielsen, 2024]
.

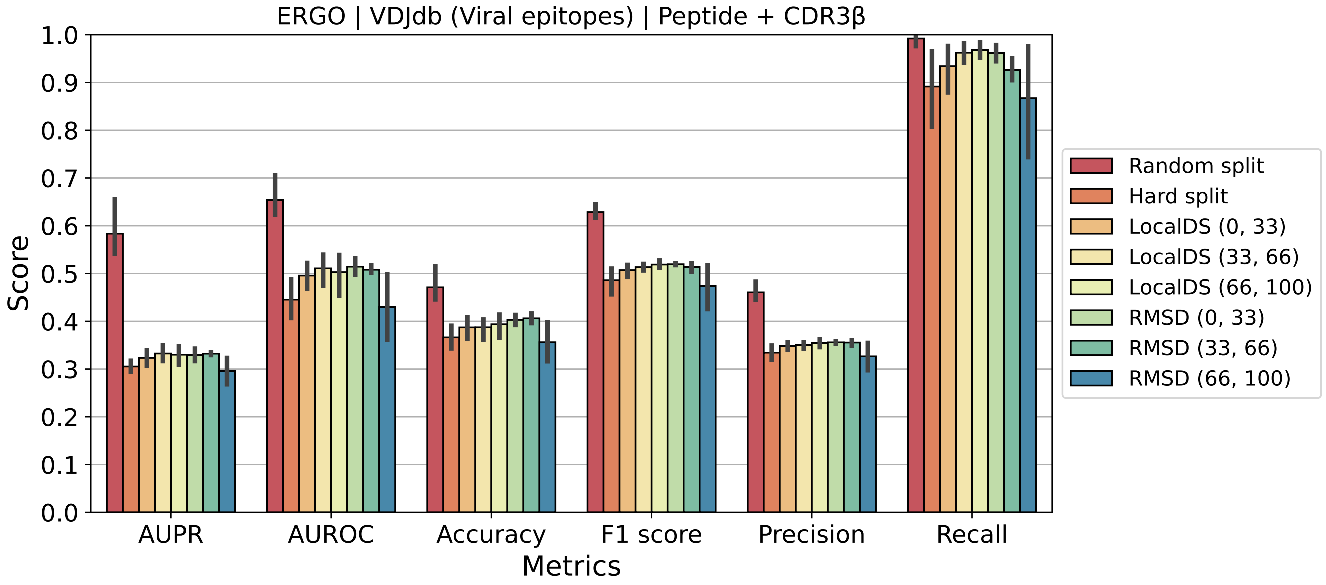

Figure S8. Performance metrics for the ERGO II model with autoencoder [Springer et al., 2021a].

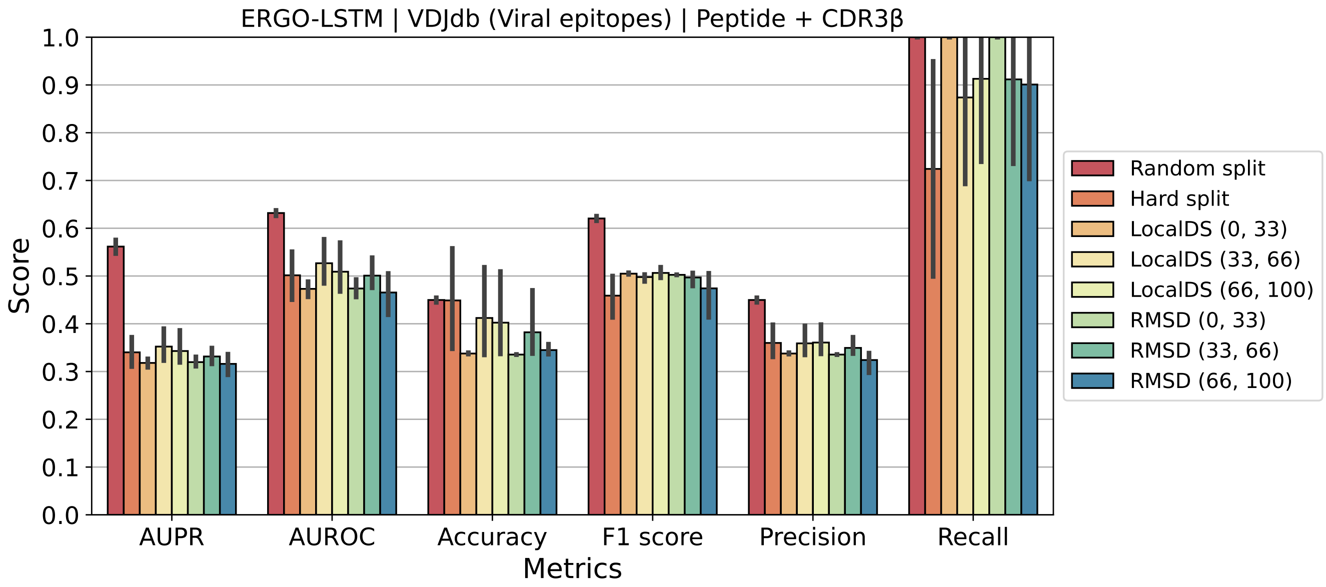

Figure S9. Performance metrics for the ERGO II model with the LSTM encoder [Springer et al., 2021a].

2.1 Macro (per-petide) Performance Scores for TCR-pMHC models

Presented below are per-peptide metrics. However, as the dataset contains many peptides with very few instances (e.g. 1-2) these metrics may not accurately represent the performance of the models.

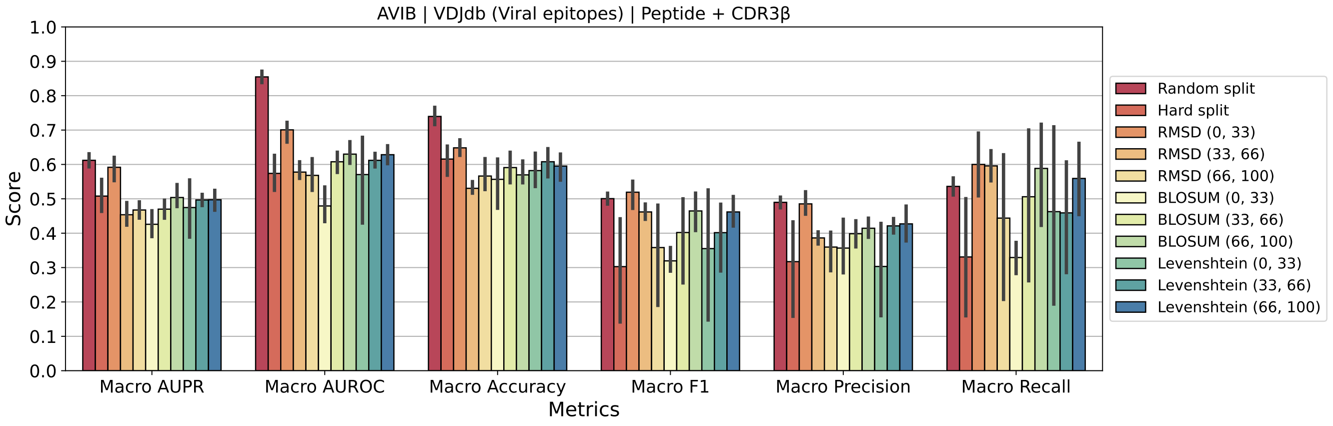

Figure S10. Macro performance metrics for the AVIB model [Grazioli et al., 2022a].

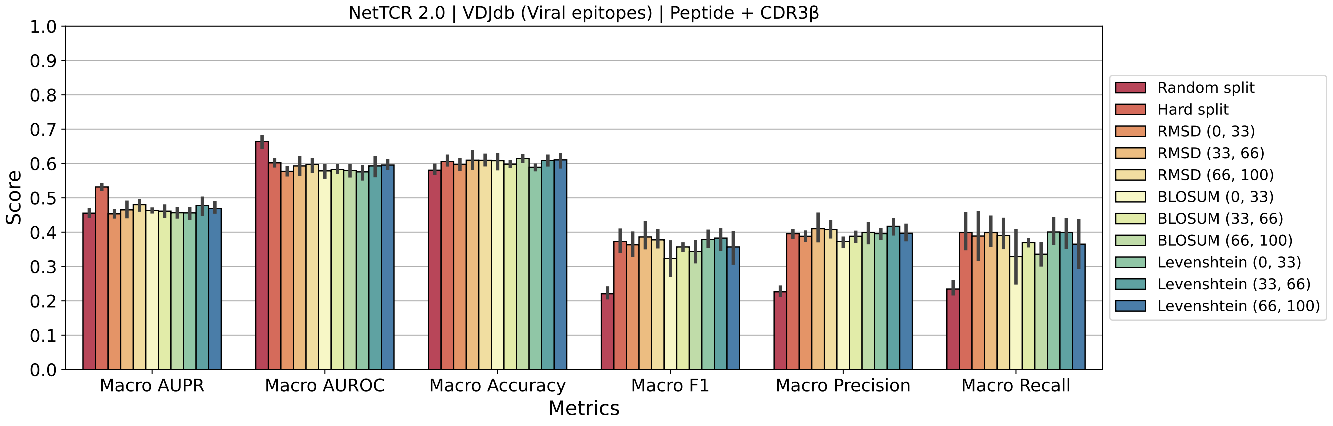

Figure S11. Macro performance metrics for the NetTCR-2.0 model [Jurtz et al., 2018].

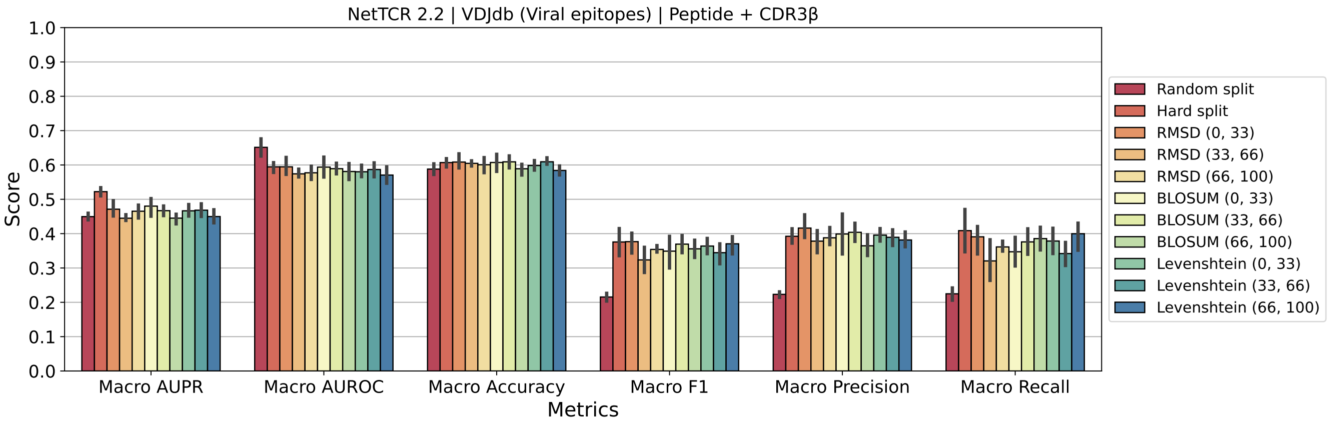

Figure S12. Macro performance metrics for the NetTCR-2.2 model [Jensen and Nielsen, 2024]
.

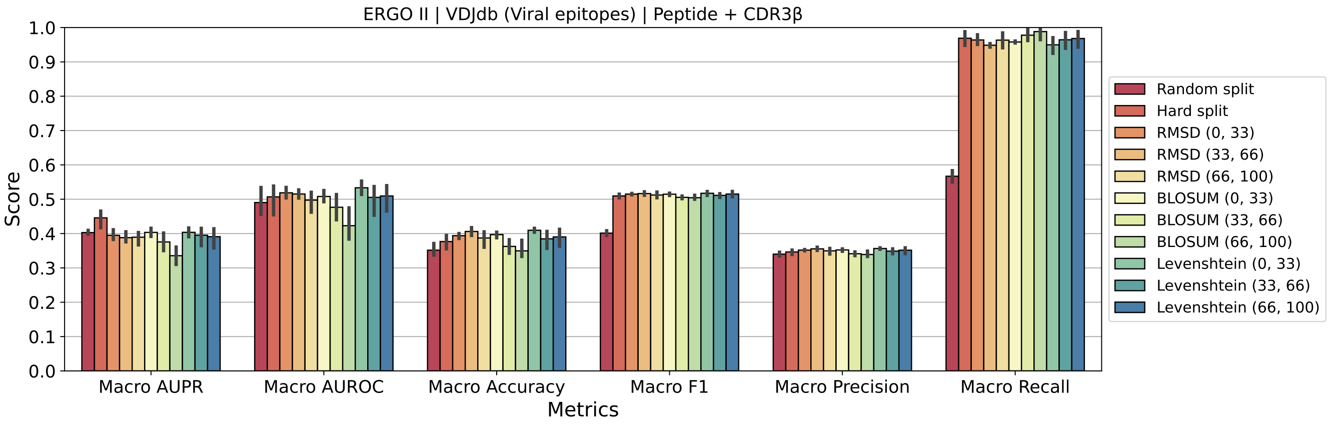

Figure S13. Macro performance metrics for the ERGO II model with autoencoder [Springer et al., 2021a].

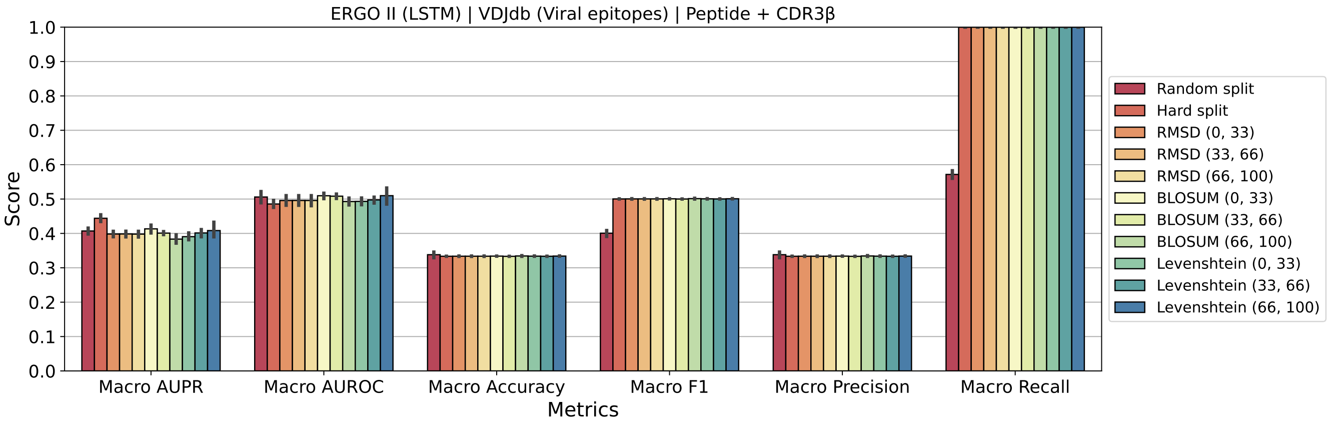

Figure S14. Macro performance metrics for the ERGO II model with the LSTM encoder [Springer et al., 2021a].

2.2 AUROC vs Difficulty Correlation

2.2.1 Sequence-based Performance

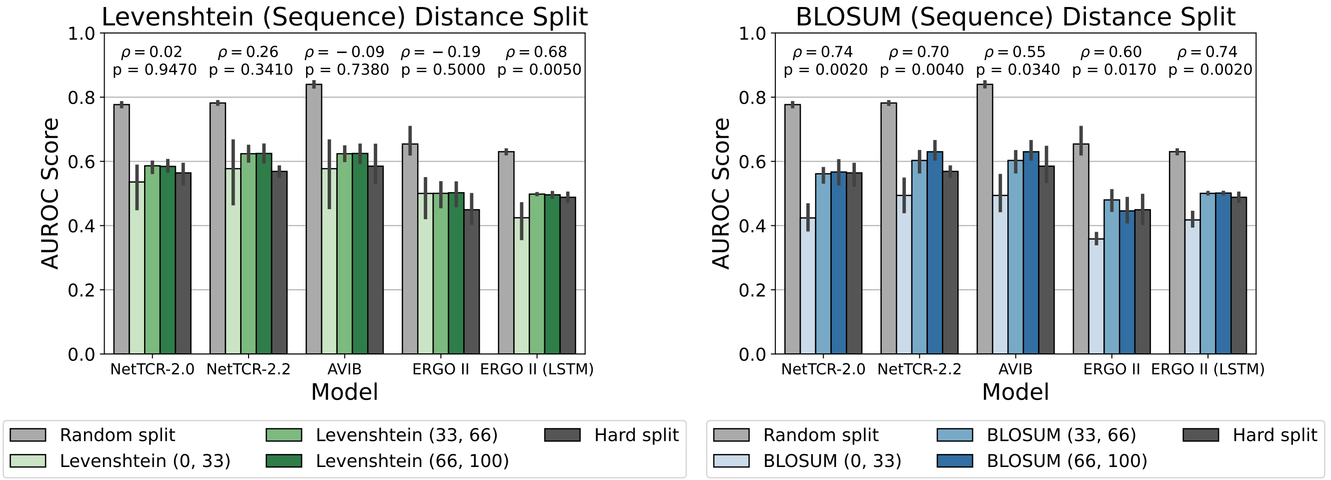

Figure S15. AUROC scores for TCR's CDR3β-peptide binding prediction for models trained and tested using various splitting techniques. The commonly used *Random Split* allows test peptides to be observed also during training. In the *Hard Split*, peptides are exclusively allocated to either the training or test set. In the *Distance Split*, we enforce specific median distances between the training and test peptides, using sequence-based distances (Levenshtein and BLOSUM). The training-test distance between peptides is controlled by selecting three percentile intervals over the cumulative median peptide-peptide distance distribution: $(0, 33)$, $(33, 66)$ and $(66, 100)$.

Spearman correlation coefficients were computed between AUROC and difficulty bins, where difficulty was defined by RMSD, BLOSUM, and Levenshtein distances overall (Table S1) and per peptide (Table S2).

2.2.2 Correlation Overall (Micro)

Model	RMSD	BLOSUM	Levenshtein
AVIB	**-0.76 (0.001)**	**0.74 (0.002)**	0.02 (0.947)
NetTCR2.0	**-0.53 (0.043)**	**0.70 (0.004)**	0.26 (0.341)
NetTCR2.2	**-0.55 (0.034)**	**0.55 (0.034)**	-0.09 (0.738)
ERGO II	-0.45 (0.089)	**0.60 (0.017)**	-0.19 (0.500)
ERGO II (LSTM)	-0.08 (0.789)	**0.74 (0.002)**	**0.68 (0.005)**

Table S1. Spearman Correlation Coefficients for Micro AUROC (with p-values in parentheses)

2.2.3 Correlation Per Peptide (Macro)

Model	RMSD	BLOSUM	Levenshtein
AVIB	**-0.70 (0.004)**	**0.76 (0.001)**	0.08 (0.789)
NetTCR2.0	0.40 (0.143)	0.08 (0.789)	0.36 (0.189)
NetTCR2.2	-0.26 (0.341)	-0.21 (0.457)	-0.17 (0.544)
ERGO II	-0.25 (0.377)	**-0.57 (0.028)**	-0.13 (0.638)
ERGO II (LSTM)	0.51 (0.052)	**-0.68 (0.005)**	**0.81 (0.000)**

Table S2. Spearman Correlation Coefficients for Macro AUROC (with p-values in parentheses)

2.2.4 Distance Split using the Min Operation

For the AVIB model, in this section we report results obtained substituting the mean operation with the min in the DS algorithm.

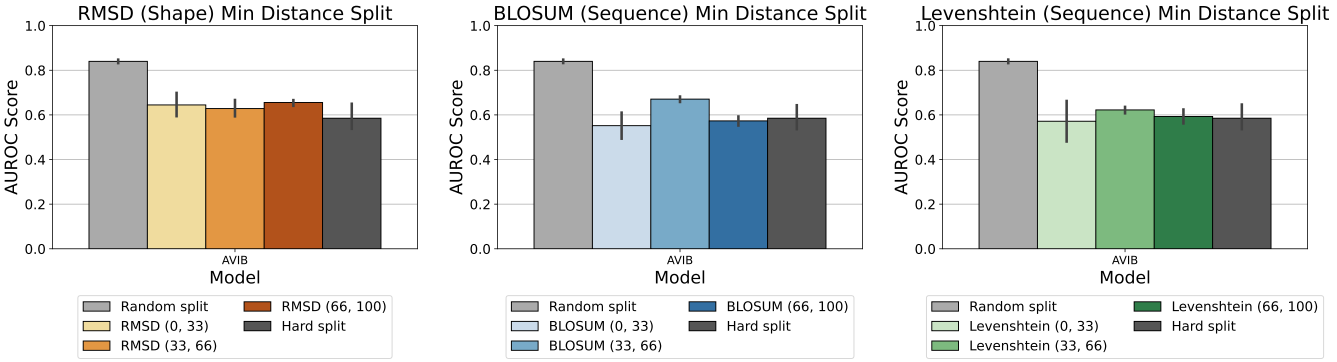

Figure S16. Distance Split using Min for RMSD, BLOSUM, and Levenshtein Distance

REFERENCES

Filippo Grazioli, Pierre Machart, Anja Mösch, Kai Li, Leonardo Castorina, Nico Pfeifer, and Martin Renqiang Min. Attentive Variational Information Bottleneck for TCR-peptide Interaction Prediction. *Bioinformatics*, page btac820, December 2022a. ISSN 1367-4803, 1367-4811. doi: 10.1093/bioinformatics/btac820. URL https://academic.oup.com/bioinformatics/advance-article/doi/10.1093/bioinformatics/btac820/6960920.

Filippo Grazioli, Anja Mösch, Pierre Machart, Kai Li, Israa Alqassem, Timothy J. O'Donnell, and Martin Renqiang Min. On TCR binding predictors failing to generalize to unseen peptides. *Front. Immunol.*, 13:1014256, October 2022b. ISSN 1664-3224. doi: 10.3389/fimmu.2022.1014256. URL https://www.frontiersin.org/articles/10.3389/fimmu.2022.1014256/full.

Mathias Fynbo Jensen and Morten Nielsen. Nettcr 2.2 - improved tcr specificity predictions by combining pan- and peptide-specific training strategies, loss-scaling and integration of sequence similarity. *eLife*

Sciences Publications, Ltd, February 2024. doi: 10.7554/elife.93934.2. URL http://dx.doi.org/10.7554/eLife.93934.2.

Vanessa Isabell Jurtz, Leon Eyrich Jessen, Amalie Kai Bentzen, Martin Closter Jespersen, Swapnil Mahajan, Randi Vita, Kamilla Kjærgaard Jensen, Paolo Marcatili, Sine Reker Hadrup, Bjoern Peters, et al. Nettcr: sequence-based prediction of tcr binding to peptide-mhc complexes using convolutional neural networks. *BioRxiv*, page 433706, 2018.

Ido Springer, Nili Tickotsky, and Yoram Louzoun. Contribution of t cell receptor alpha and beta cdr3, mhc typing, v and j genes to peptide binding prediction. *Frontiers in Immunology*, 12, 2021a. ISSN 1664-3224. doi: 10.3389/fimmu.2021.664514. URL https://www.frontiersin.org/journals/immunology/articles/10.3389/fimmu.2021.664514.

Ido Springer, Nili Tickotsky, and Yoram Louzoun. Contribution of t cell receptor alpha and beta cdr3, mhc typing, v and j genes to peptide binding prediction. *Frontiers in immunology*, 12:664514, 2021b.
