# Supplementary material for: Assessing the generalization capabilities of TCR binding predictors via peptide distance analysis
Source: PLoS One. 2025 May 20;20(5):e0324011. doi: 10.1371/journal.pone.0324011 (PMC12091837; doi:10.1371/journal.pone.0324011)
Supplement: Attachment — Submitted filename: _NEC__Distance_Split_letter.pdf [file pone.0324011.s002.pdf]

Assessing the Generalization Capabilities of TCR Binding Predictors via Peptide Distance Analysis

Response to Reviewers

October 18, 2024

Dear Dr. Magallanes, dear Reviewers,

We would like to express our sincere gratitude for your thoughtful and constructive feedback on our manuscript. We have carefully considered all the reviewers' comments and have made revisions to address each point. Generally, we expanded the paper to have more TCR-pMHC models to ensure the robustness of results. Additionally, we removed LocalDS because the way it was implemented was not weighing the score by BLOSUM weights. Instead, we added Levenshtein distance as a baseline, and BLOSUM score (obtained after an alignment) to add more physico-chemical information.
Below, we provide detailed responses to each of the comments and explain the corresponding changes made in the manuscript.

Reviewer 1

The authors introduce a novel approach to assess the generalisation capabilities of TCR-pMHC binding predictors through the Distance Split (DS) method. This technique evaluates machine learning (ML) models, such as the Attentive Variational Information Bottleneck (AVIB) and NetTCR-2.0, using both sequence-based and structural (3D) metrics. They focus on the importance of structural metrics, leveraging advances in protein modeling with third-party tools like ESMFold and OmegaFold to enhance the prediction accuracy of ML models. This study emphasizes the necessity for robust evaluation methods that consider 3D structural distances to predict the real-world applicability of TCR-pMHC interaction models more reliably.
The motivation and objective of the research are founded on a solid and clear background, making the concept of the paper highly comprehensible and appreciable. However, there are several aspects of the methodology, particularly the new split method, which could benefit from further clarification and justification. The framework for justifying this new method seems to require more robust reasoning to convincingly support the claims made. Given these concerns, I would suggest substantial revisions to address these points before reconsidering the paper for publication.

Major

Comment 1 - Dataset Split Evaluation

The paper discusses the relationship between performance decline and distance magnitude when using the DS algorithm with structural distance. It should be more insightful to include comparisons across models trained on different splits, such as DS versus HS, and evaluated on a common ground truth test set that is created via DS or HS. In short, when stating the usefulness of the dataset split based on the structural distance, it is not enough to show only the performance decline versus the distance magnitude, probably. This would provide a clearer picture of the effectiveness of each splitting method when tackling a real-world scenario, where researchers train models on whatever the split is and use the models on the unknown test set which might or might not be structurally similar to the training set.

Response 1

We appreciate the reviewer's careful comment on this central aspect of the testing methodology. However, we believe there may have been a misunderstanding regarding the interpretation of the splits, e.g., DS. To clarify, our intention was to employ different splitting techniques to create controlled test sets, in which test peptides are e.g. at a given distance from the training ones. This allows to verify our hypothesis on whether models generalise better when test peptides are "closer" to the training ones, according to a given metric. Having a common test set, as suggested by the reviewer, defies the purpose of our experiments. We have now revised the text to make this explanation clearer and avoid any further confusion.
Regarding the performance decline, we have also added a spearman correlation of the performance against difficulty for each model and distance.

Comment 2.1 - Clarification of DS Algorithm

The DS algorithm's definition and explanation would benefit from being included in the main body of the paper rather than the Supplementary Materials because it is the main part of the research.

Response 2.1

We have now moved the Distance Split Algorithm to the main text.

Comment 2.2

A more detailed description could address potential ambiguities, such as whether the use of median distance might still allow for overlap in peptide characteristics between the training and test sets. I mean, if it takes the median of the distance per peptide when setting a test set, does it still contain a chance of having similar peptide set, similar with training? Perhaps considering the minimum distance per peptide for splitting would offer a stricter and potentially more effective division after creating the vector M for each peptide.

Response 2.2

As suggested by the reviewer, the minimum distance would indeed be a more stringent separation criterion, so we addressed this in the discussion of the paper. Analogously, we could have employed a more granular binning for the median, e.g., the 90-100 percentile interval, instead of 66-100. Unfortunately, due to the small dataset size, more stringent separation between training and test would lead to test sets which are too small to provide robust assessment of the model performance.

Comment 2.3

The methodology for sampling peptides for the test and validation sets raises concerns regarding potential overfitting, especially because the validation data seems too similar to the test data. Clarifying the strategy behind this choice and considering the implications of such a setup in real-world scenarios would be beneficial.

Response 2.3

The test and validation peptides are always different. We first operate an outer split, which gives us the test set. From the remaining data points, we operate an inner split, which gives us the validation and training sets. When using the DS, training, validation and test peptide sets are always disjoint. Hence, there cannot be sequence memorisation. The same holds for the HS (but nor for RS). The aim of the validation set in this study is to estimate the generalization performance on the test set. As such, it is important that they both come from a similar distribution and that the peptides are unique for each set. Therefore, there is no risk of overfitting, as also shown by the significantly lower performance compared to the RS. We thank the reviewer for pointing this out, and improved the text clarity.

Comment 2.4

What is a leeway factor l and what is the budget and What is the sN of N_{test} relationship of them?

Response 2.4

To improve clarity, we have divided the algorithm into sections, added more comments, and simplified the terminology (see Algorithm 1 in main text).
Budget: if a user needs to build a training and test set, they will specify a percentage split, for example 80-20, which multiplied by the total number of data points define a budget. For example, for 1000 total data points, the budget would be 800 for training and 200 for testing.
Leeway factor l: It is possible that there are not enough peptides to simultaneously fill the test budget and fulfil the distance constraints. The leeway factor is a percentage that allows us to define a "leeway" on the requested budget. If a user requests 900 data points, a leeway factor of 0.05, would allow the creation of a set with 855 samples if no more data points are available (see while condition in the Split Algorithm).
sN and N_{test}: sN stood for test split ratio $\times$ Number of samples.

Comment 3

Are the two benchmark models based on the sequence? If so, it was remarkable to observe that the longer distance split did not change the AUC. If there is a way to include structural features in peptide, do you think the longer distance of RMSD did not affect the AUC?

Response 3

All models input the amino acid sequences, meaning that no structural information is added. We have now expanded the selection of models to include NetTCR-2.2, ERGO II and ERGO II (LSTM), as requested. As structural information may provide important features about the binding prediction problem, the inclusion of peptide and TCR structures should increase the robustness and accuracy of TCR-pMHC models. This was shown a year after our original manuscript submission by Pham et al. [2024], that also use 3D structures estimated by OmegaFold, and Ji et al. [2024] that use AlphaFold2. For completeness, we added references to these two papers in our manuscript. However, the DS algorithm should still work with the addition of structural features. This is supported by the BLOSUM DS splits, where sequence-based models exhibit strong correlations between difficulty and performance.

Comment 4.1 - Peptide Distance

The method of calculating distances might be impacted by peptide length variations. Including information on peptide lengths and discussing how they affect distance calculations would provide a more thorough understanding of the model's applicability.

Response 4.1

Indeed, RMSD may be impacted by the peptide length. Normalisation methods such as RMSD_100 allow normalisation of RMSD only when the number of atoms of both proteins is equal. In the case of viral epitopes, however, the length might vary between 8 and 15 (see Table 1 below). In this case however, the proteins are so small that it would likely not lead to significant differences in distance. Additionally, for the number of peptide pairs analyzed, the comparison with a matching sequence

length is given by:

$$\begin{aligned}\text{Matching Length Pairs} &= \sum_i \frac{n_i(n_i-1)}{2} \\ &= \frac{10(10-1)}{2} + \frac{705(705-1)}{2} + \frac{29(29-1)}{2} \\ &+ \frac{6(6-1)}{2} + \frac{2(2-1)}{2} + \frac{1(1-1)}{2} \\ &+ \frac{3(3-1)}{2}\end{aligned}$$

This results in a total of 248,630 matching length pairs comparisons out of 285,090 (about 87%), meaning that most of the comparison is based on ”same-length” peptides. We updated the discussion to explain the shortcomings of RMSD in the cases where the proteins have unequal number of amino acids.

Number of Amino Acids	Count
8	10
9	705
10	29
11	6
12	2
13	1
15	3

Table 1: Number of amino acids of epitopes and corresponding counts in the viral peptide dataset

Comment 4.2

Additionally, is α Carbon enough to model the peptide-peptide similarity? Often the case, side chains of amino acid form the bond with other molecules.

Response 4.2

In the field of proteins, RMSD is used as a method of comparison. Folding models like AlphaFold, ESMFold, and OmegaFold have no physical constraints imposed on the side-chains, meaning that they may (and very often will) generate impossible conformations of side-chains (e.g. some with overlapping atoms) Moore et al. [2022]. Using α Carbon backbone avoids physical artifacts generated by models and is usually more computationally efficient and similarly accurate Mechelke and Habeck [2010]. An alternative would be to run physical simulations to relax the peptide, however, given the small size it may not reflect the actual conformation of the peptide inside the MHC. Regarding bonds, the only side-chain able to form a physical bond is Cysteine for disulphide bridges. If this were to happen in a peptide, it would be reflected in its α Carbon positioning.

Minor

1) In evaluating TCR-pMHC these papers are good to cite, https://doi.org/10.3389/fimmu.2020.01803, https://doi.org/10.3389/fbinf.2023.1274599, 10.1186/s12859-019-3109-6

- We added these papers in the main text to strengthen the introduction section.

2) Line9: what does this mean by the word ”on ML model risk assessments” in the Abstract?

- We refer to the concept of empirical risk borrowed from statistical learning
- See: https://en.wikipedia.org/wiki/Empirical_risk_minimization

3) Line 176: the citation of the benchmark model is inconsistent. "Jurtz et al. (2018)" and "(Grazioli et al., 2022b)". The end of sentence is unclear.

- We have clarified that the citation (Grazioli et al., 2022b) refers to the AVIB model and the Jurtz et al. (2018) refers to NetTCR2.0.

4) Line 177: "This model presents probabilistic encoders". Which model is it?

- The AVIB model. We have clarified in section 2.3 each of the models used and added a brief description of models features.

5) In Table 1, the average of peptide counts is 45, but why is it not "87.1 = 65,946 / 757"

- Our dataset contains 34,415 binding samples. As there is a lack of "non-binding" samples, we generated 36,641 non-binding samples which sum up to 65,946. The correct average number of samples is therefore 34,415 / 757 $\approx$ 45.462 as presented in Table 1.

6) Line 200: why are there 5 different random seeds? Is there any randomness in DS splits?

- There is randomness in the DS as shown in the Algorithm 1 in the main text. Specifically, first, a peptide is randomly sampled, then, it is included in the test set if its number of (TCRβ, peptide) pair counts plus the pair counts already in the test set are within the desired test budget. If not, the peptide is discarded and a new one is selected. This sampling procedure is stochastic and controlled by a random seed.

7) Do some of the metrics need thresholds of the confidence values in Figure S2?

- The metrics presented in Figure S2 present standard deviation as error bars.

8) There is a missing period in the caption of Figure 4.

- Rectified.

Reviewer 2

The manuscript presents an interesting and important aspect of unseen epitope binding prediction (to TCRs): structure based train val test splitting instead of unique or sequence similarity based. The authors show that the the structural distance could be used better than sequence based distances for uncertainty estimates. However, the current manuscript is not thorough enough. The current way of presenting does not present distance based uncertainty (which would be very valuable i.e. this peptide is too distance the model cannot evaluate the binding probability, also quite hard) of a new peptide rather than generally structural differences are more important than sequence identity.

Major

Comment 1 - More (diverse) methods in the comparison

There are only two methods presented and they use the same amino acid representation and similar architecture. To add more trustworthiness for the claims it would be important to add more methods that use different architectures and input representations. (Also NetTCR2 is used instead of the newer version NetTCR2.2)

Response 1

We have added additional models to our manuscript: NetTCR2.2, ERGO II (autoencoder), and ERGO II (LSTM encoder). AVIB and the NetTCR models employ BLOSUM-encoded amino acid sequences, while ERGO models employ learned embedding vectors of the amino acid sequences.

Comment 2.1

What is the importance of the new splitting why not use unseen epitope test (HS) and then bin by distances like done in e.g. ImRex, EPIC-TRACE, MixTCRpred

Response 2.1

In the EPIC-TRACE publication, epitopes appear to be binned by number of instances in the dataset, not by distance (see EPIC-TRACE Publication Results Section and Figure 3). ImRex employs a similar strategy to the Hard Split followed by binning on edit distance (see Publication section "Strong performance for closely related epitopes"). MixTCRpred seems to use a similar edit distance (as seen in the code: here).
In our paper, we use more biologically relevant distances, such as BLOSUM and 3D shape RMSD. On the other hand, there is no guarantee that the "unseen epitopes" will not be very similar to the training ones. Meaning that binning by distance on unseen epitopes does not guarantee that they will be at a certain distance, i.e., they could all be similar to the training ones. With the distance split algorithm, we could define the bin of the distance beforehand, so that the resulting test peptides are guaranteed to be at a certain distance and to contain the desired budget of data points.

Comment 2.2

An addition of epitope score binning (and correlation) would be a valuable addition to verify the results. (also by using different binning methods on HS many methods can be analyzed with same computational cost of training the models/ or more seeds can be evaluated)

Response 2.2

We have added Spearman correlations and their p value in Figure 3 of the main paper and Supplementary Section 2.2. As per advice from the reviewer, we have also added the correlations per peptide.

Comment 3

The DS seems to control only for datapoint number but not number of peptides, which feels quite important in this case as the test amount can be filled with 1 or with many peptides

Response 3

Some epitopes are more commonly studied than others, meaning they are over-represented in terms of (peptide, TCR) pairs. Controlling for the number of peptides will not guarantee that the number of (peptide, TCR) pairs are filled for the different splits, potentially resulting in too large test sets and too small training sets (or vice versa). However, as shown in Figure 2 the number of peptides in each split generated by the DS algorithm is roughly consistent.

Comment 4.1

Did you try DS with min distance, or mean, or mean of min X e.g. 10? What is the reasoning behind the median?

Response 4.1

The DS algorithm is compatible with all of these strategies. We chose to use the median because the data is heavily skewed towards specific epitopes, thus the inclusion of a single epitope could end up affecting the mean massively. These situations are common in nature when new viruses arise and are studied (for example COVID-19) and more datapoints are added to public datasets.

Comment 4.2

What are the advantages of splitting versus binning on HS?

Response 4.2

Binning on HS does not guarantee that the training and the test/val split have a certain distance. In the worst-case scenario, the HS could be filled with very similar peptides to the training ones. Thus, the difference in performance between bins of the HS would be minimal. With the hard split, the train, test, and validation sets are built progressively to respect the distance requested by the user, giving more control over the difficulty of the split and the desired distance.

Minor

1) Add String edit distance (Levenshtein) as a simple baseline and maybe some physicochemical property distance or other distance that is different from sequence and structure

- We have replaced LocalDS distance with BLOSUM62 distance, which is a substitution matrix indicating the log likelihood of a change of amino acids meaning it takes into account physicochemical properties of the amino acids. For instance, a change between a large hydrophobic amino acid (Trp) to a smaller hydrophillic one (Ser) is very unlikely (high distance).
- We have also added Levenshtein distance, as suggested.

2) Regarding Figure 1/2, the most abundant epitopes are not shown in the figures as the distribution of datapoints per epitope is unbalanced and some epitopes have thousands of datapoints whereas most have only a few. This is somewhat confusing for the interpretability of the plot

- Indeed, due to the skewed nature of the dataset, the most abundant epitopes are not shown. We have plotted the figure without limiting the peptide count axis, however, the plot is not as meaningful (See Fig. 1)

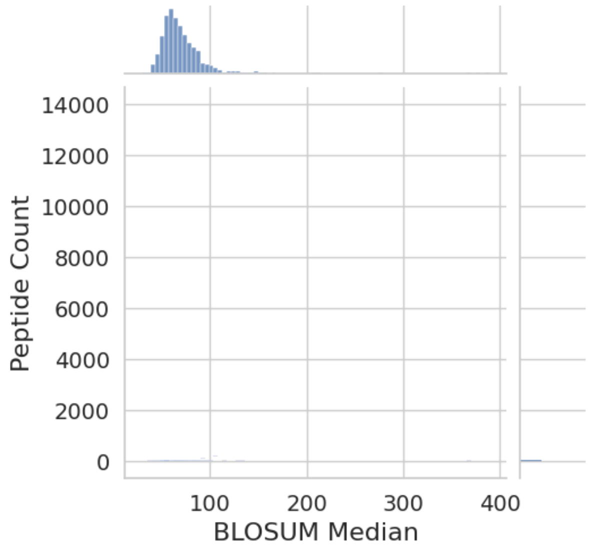

Figure 1: Peptide Count vs BLOSUM

3) Also why is the mean plotted while the median is used in in the split?

- As suggested we have updated the plots in the figure to display the median.

4) I believe that the performance should be for peptides not for datapoints thus choosing macro (separately for each peptide and then average) over micro (over all datapoints directly) AUROC could be considered

- As per reviewer's request, we have now added the Macro (per-peptide) metrics in the Supplementary Section 2.1 . It is worth noting, however, the dataset is so skewed that some peptides only have one instance, meaning that some of the metrics, for example AUROC, may be undefined for those samples. This leads to performance metrics mainly based on more frequent peptides.

5) Number of unique test peptides (mean) in each split would also be informative (even more so when binning HS and/or using macro AUROC)

- We have added the plot of the mean number of peptide and standard deviation to the Supplementary Material and here below (see Figure 2)

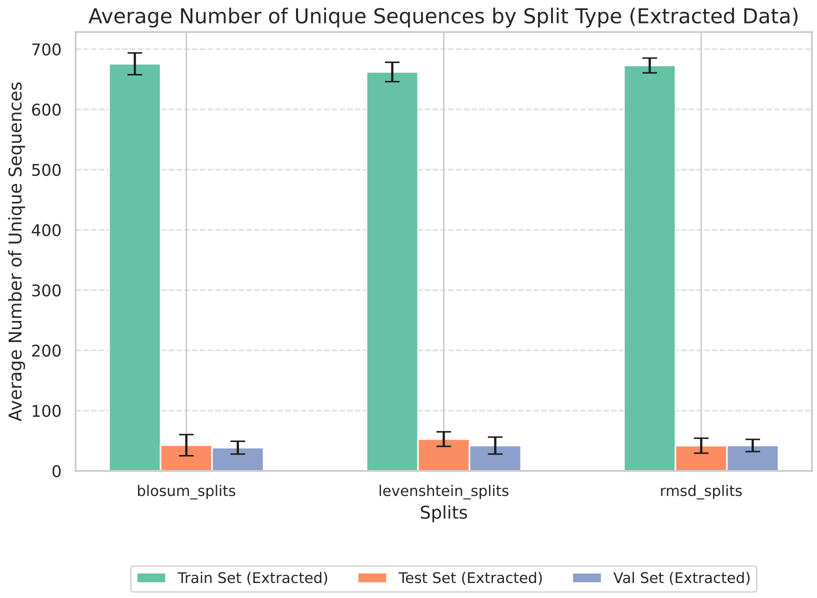

Figure 2: Number of Peptides per Split Type

6) Also, check this out. Cannot require the comparison to a method that was published after submission, but this paper seems to have same direction results, improving prediction performance by using the epitope structure: "Hongchen Ji, Xiang-Xu Wang, Qiong Zhang, Chengkai Zhang, Hong-Mei Zhang, Predicting TCR sequences for unseen antigen epitopes using structural and sequence features, Briefings in Bioinformatics, Volume 25, Issue 3, May 2024, bbae210, https://doi.org/10.1093/bib/bbae210"

- For completenes, we have added a reference to this paper and other similar papers that were published on the topic after our original manuscript.

Sincerely,
The authors

References

Hongchen Ji, Xiang-Xu Wang, Qiong Zhang, Chengkai Zhang, and Hong-Mei Zhang. Predicting TCR sequences for unseen antigen epitopes using structural and sequence features. *Briefings in Bioinformatics*, 25(3):bbae210, 05 2024. ISSN 1477-4054. doi: 10.1093/bib/bbae210. URL https://doi.org/10.1093/bib/bbae210.

Martin Mechelke and Michael Habeck. Robust probabilistic superposition and comparison of protein structures. *BMC Bioinformatics*, 11(1), July 2010. ISSN 1471-2105. doi: 10.1186/1471-2105-11-363. URL http://dx.doi.org/10.1186/1471-2105-11-363.

Peter B. Moore, Wayne A. Hendrickson, Richard Henderson, and Axel T. Brunger. The protein-folding problem: Not yet solved. *Science*, 375(6580):507–507, February 2022. ISSN 1095-9203. doi: 10.1126/science.abn9422. URL http://dx.doi.org/10.1126/science.abn9422.

My-Diem Nguyen Pham, Chinh Tran-To Su, Thanh-Nhan Nguyen, Hoai-Nghia Nguyen, Dinh Duy An Nguyen, Hoa Giang, Dinh-Thuc Nguyen, Minh-Duy Phan, and Vy Nguyen. epitcr-kda: Knowledge distillation model on dihedral angles for tcr-peptide prediction. *bioRxiv*, 2024. doi: 10.1101/2024.05.18.594806. URL https://www.biorxiv.org/content/early/2024/05/21/2024.05.18.594806.
